# Antipsychotic medication for women with schizophrenia spectrum disorders

**DOI:** 10.1017/S0033291721004591

**Published:** 2022-03

**Authors:** Bodyl A. Brand, Yudith R. A. Haveman, Franciska de Beer, Janna N. de Boer, Paola Dazzan, Iris E. C. Sommer

**Affiliations:** 1 Department of Biomedical Sciences of Cells & Systems, Section Cognitive Neurosciences, University Medical Center Groningen, University of Groningen, Groningen, the Netherlands; 2 Department of Psychiatry, UMC Utrecht Brain Center, University Medical Center Utrecht, Utrecht University, Utrecht, the Netherlands; 3 Department of Psychological Medicine, Institute of Psychiatry, Psychology and Neuroscience, King's College London, London, UK; 4 National Institute for Health Research (NIHR) Mental Health Biomedical Research Centre at South London and Maudsley NHS Foundation Trust and King's College London, London, UK

**Keywords:** Antipsychotic treatment, dopamine sensitivity, pharmacodynamics, pharmacokinetics, psychosis, schizophrenia, sex differences

## Abstract

There are significant differences between men and women in the efficacy and tolerability of antipsychotic drugs. Here, we provide a comprehensive overview of what is currently known about the pharmacokinetics and pharmacodynamics of antipsychotics in women with schizophrenia spectrum disorders (SSDs) and translate these insights into considerations for clinical practice. Slower drug absorption, metabolism and excretion in women all lead to higher plasma levels, which increase the risk for side-effects. Moreover, women reach higher dopamine receptor occupancy compared to men at similar serum levels, since oestrogens increase dopamine sensitivity. As current treatment guidelines are based on studies predominantly conducted in men, women are likely to be overmedicated by default. The risk of overmedicating generally increases when sex hormone levels are high (e.g. during ovulation and gestation), whereas higher doses may be required during low-hormonal phases (e.g. during menstruation and menopause). For premenopausal women, with the exceptions of quetiapine and lurasidone, doses of antipsychotics should be lower with largest adjustments required for olanzapine. Clinicians should be wary of side-effects that are particularly harmful in women, such as hyperprolactinaemia which can cause oestrogen deficiency and metabolic symptoms that may cause cardiovascular diseases. Given the protective effects of oestrogens on the course of SSD, oestrogen replacement therapy should be considered for postmenopausal patients, who are more vulnerable to side-effects and yet require higher dosages of most antipsychotics to reach similar efficacy. In conclusion, there is a need for tailored, female-specific prescription guidelines, which take into account adjustments required across different phases of life.

## Introduction

It was long thought that women with a schizophrenia spectrum disorder (SSD) had a more favourable course compared to male patients, but that idea should be reconsidered. Female patients have the same number of readmissions during their lifetime (Ceskova, Prikryl, Libiger, Svancara, & Jarkovsky, 2015; Sommer, Tiihonen, van Mourik, Tanskanen, & Taipale, 2020), have similar recovery rates (12.9% for women and 12.1% for men) (Jaaskelainen et al., 2013) and similar functional outcomes 10 years after a first psychotic episode (Ayesa-Arriola et al., 2020; Mayston et al., 2020). Although male patients experience more negative symptoms, females have more affective symptoms (Ochoa, Usall, Cobo, Labad, & Kulkarni, 2012). Aggression towards others is more common in males, whereas self-harm and suicide attempts are more common among female patients (Dama et al., 2019; Dubreucq et al., 2021; Jongsma, Turner, Kirkbride, & Jones, 2019; Sommer et al., 2020). Although the later age of onset and lower comorbidity with substance abuse in women may lead to better functioning in the early stages of the illness (Usall, Ochoa, Araya, & Márquez, 2003), this benefit is not maintained in the more chronic phase and advantages for women even seem to reverse after the age of 50 (Mayston et al., 2020; Shlomi Polachek et al., 2017; Thorup, Waltoft, Pedersen, Mortensen, & Nordentoft, 2007). Hence, the idea that women have a better overall course of SSD compared to men is not correct. However, the vast majority of the literature and guidelines on antipsychotic treatment neglects differences between male and female patients and base their conclusions on studies with predominantly male participants (Huhn et al., 2019; Lally & MacCabe, 2015; Lange, Mueller, Leweke, & Bumb, 2017; Phillips & Hamberg, 2016; Santos-Casado & García-Avello, 2019; Zakiniaeiz, Cosgrove, Potenza, & Mazure, 2016).

Psychosocial differences in diet, smoking and substance abuse between men and women (i.e. gender differences), can influence the efficacy and tolerability of antipsychotics (Seeman, 2004). In addition, biologically determined differences (i.e. sex differences), such as body composition and hormonal transitions affect the drug pharmacokinetics, determined by the absorption, distribution, metabolism and excretion, and drug pharmacodynamics which involves receptor binding, receptor sensitivity and the receptor binding profile of a drug (Iversen et al., 2018; Lange et al., 2017; Zucker & Prendergast, 2020). The female sex hormones in general, and oestrogens in particular play a major role in these sex differences (Brand, de Boer, & Sommer, 2021; González-Rodríguez & Seeman, 2019). However, current guidelines on the prescription of antipsychotics do not take these differences in account (Keepers et al., 2020; Ventriglio et al., 2020). This review serves as a comprehensive overview of currently available literature on the differences in the action of antipsychotic medication in female *v.* male patients with SSD, with special considerations for female-specific pharmacotherapy regimens during different (hormonal) stages of life (i.e. menarche, pregnancy, lactation and menopause).

## Gender differences in prescription

Although some patients prefer to be treated without medication, most psychotic episodes require pharmacological treatment with antipsychotics. Although classical antipsychotics such as haloperidol are still being used as second generation and other newer antipsychotics such as aripiprazole, risperidone, olanzapine, amisulpride and quetiapine are often preferred by psychiatrists given their better efficacy–tolerability balance (Kahn et al., 2008). Clozapine is usually provided as a second or third line of treatment, when two other antipsychotics did not yield remission from psychosis. Although clozapine is superior in efficacy for these individuals, it does have some bothersome side-effects, such as being diabetogenic, inducing weight gain, severe constipation and (rarely) agranulocytosis (Nielsen, Damkier, Lublin, & Taylor, 2011).

Although no sex-specific guidelines currently exist for prescribing antipsychotics, notable differences are observed in prescription patterns between men and women, caused by preferences of both the physician and the patient. Women may experience some side-effects as more severe than men and may, for example, encounter more difficulties with antipsychotic-induced weight gain (Achtyes et al., 2018; Connors & Casey, 2006). The risk of weight gain is, in particular, of great influence in the decision to take medication in women, causing non-adherence to prescribed medications specifically in this gender (Achtyes et al., 2018; Lambert et al., 2004).

Based on a Finnish nation-wide cohort study, women are more often prescribed quetiapine and aripiprazole, whereas men are more often prescribed clozapine and olanzapine (Sommer et al., 2020). Also, the use of additional psychotropic medication (e.g. antidepressants, mood stabilisers and benzodiazepines) is more common in women compared to in men (Ceskova & Prikryl, 2012; Sommer et al., 2020). In the USA and European countries, prescription rates of long-acting injectable (LAI) antipsychotics are lower in women (Arnold et al., 2004; Mahadun & Marshall, 2008; Shi et al., 2007; Sommer et al., 2020), although the compliance to medication is similar in men and women (Caqueo-Urízar, Fond, Urzúa, & Boyer, 2018; Castberg, Westin, & Spigset, 2009; Leijala, Kampman, Suvisaari, & Eskelinen, 2021).

## Sex differences in the pharmacokinetics of antipsychotics

**Fig. 1.**
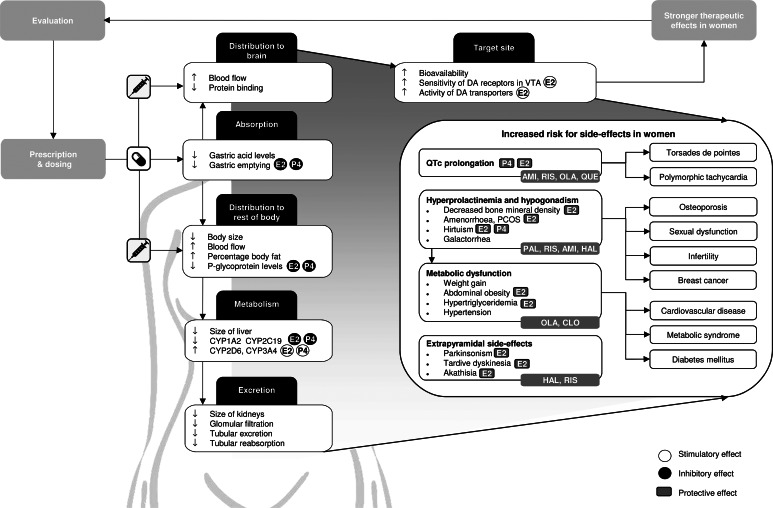
Graphical overview of relevant differences in pharmacokinetics, pharmacodynamics and therapeutic effects of antipsychotics in women compared to men. Side-effects that should be obtaining additional attention in women are defined, together with commonly used antipsychotics that carry the highest risks for these side-effects. In addition, the stimulatory and inhibitory effects of oestradiol (E2), which is the predominant form of oestrogen, and progesterone (P4) on pharmacokinetic processes are shown, as well as the protective effects of E2 and P4 on specific side-effects. CYP, cytochrome P450; AMI, amisulpride; CLO, clozapine; HAL, haloperidol; OLA, olanzapine; PAL, paliperidone; QUE, quetiapine; RIS, risperidone; E2, oestradiol; P4, progesterone; DA, dopamine; VTA, ventral tegmental area.

Body composition differs significantly between the sexes: women have smaller organs, more fatty tissue and less muscle tissue, which changes the volume of distribution, especially for lipophilic drugs (Seeman, 2018). In addition, women have some 10–15% greater blood flow to the brain, which makes it easier for a drug to reach their target receptor (Fig. 1) (Gur & Gur, 1990). Castberg, Westin, Skogvoll, and Spigset (2017) included 43 079 blood samples of patients between 18 and 100 years old using olanzapine, risperidone or quetiapine and concluded that women generally have 20–30% higher dose-adjusted concentrations, which is a proxy for bioavailability, compared to men. Another study, including 26 388 patients of all ages, reported higher dose-adjusted concentrations in women for 11 out of 12 antipsychotics (Jönsson, Spigset, & Reis, 2019), with the largest differences for olanzapine and clozapine (59.0% and 40.4% higher in women, respectively), whereas for quetiapine, dose-adjusted concentrations were 6.4% lower in women. Similarly, Eugene and Masiak (2017) reported dopamine receptor occupancy rates of 70% for male and female patients (mean age: 25.6 ± 7.9 for men; 28.9 ± 9.1 for women), whereas men were taking a higher dose (20 *v.* 10 mg/day). Since women are often treated with multiple psychotropic medications (Sommer et al., 2020), drug interactions are especially relevant for this sex and become even more crucial when women are overmedicated. For example, clinically relevant effects on serum levels of several antipsychotics have been reported for the selective serotonin reuptake inhibitors (SSRIs) fluoxetine, fluvoxamine and paroxetine (Spina & De Leon, 2014). In addition, sex differences in the pharmacokinetics of antidepressants should be taken into account in women with SSD who are also being treated for depression, which are discussed in detail in a review by Damoiseaux, Proost, Jiawan, and Melgert (2014).

### Absorption and distribution

When administered orally, the absorption of a drug largely depends on gut physiology. Women's stomachs are on average less acidic compared to men's and this increases the absorption of antipsychotic drugs (Soldin & Mattison, 2009; Walters & Levitan, 2020). Additionally, female sex hormones reduce gastric emptying and intestinal motility (Freire, Basit, Choudhary, Piong, & Merchant, 2011), resulting in lower gastrointestinal transit rates in women (Hutson, Roehrkasse, & Wald, 1989; Jiang, Greenwood-Van Meerveld, Johnson, & Travagli, 2019). This enhances drug absorption and increases the bioavailability of oral antipsychotics (Stillhart et al., 2020). After menopause, transit rates increase to a level similar to that of age-matched men (Camilleri, 2020; Gonzalez, Loganathan, Sarosiek, & McCallum, 2020).

The absorption and distribution of antipsychotic drugs is influenced by P-glycoprotein (P-gp), an efflux transporter that is located on the cell membrane that limits the systemic exposure of its substrates (Elmeliegy, Vourvahis, Guo, & Wang, 2020). P-gp levels are some two-fold lower in women compared to that in men, being decreased by oestrogens and progesterone (Bebawy & Chetty, 2009; Nicolas, Espie, & Molimard, 2009). Although lower P-gp levels thus enable a drug to enter the brain more easily, non-target organs also become more accessible for the drug, potentially causing more side-effects (Fig. 1) (Hiemke et al., 2018). Sex differences in bioavailability of antipsychotics are expected to be more pronounced for drugs that bind P-gp more tightly (e.g. risperidone and aripiprazole) (Table 1) (Bebawy & Chetty, 2009; Doran et al., 2005; Linnet & Ejsing, 2008; Moons, De Roo, Claes, & Dom, 2011; Nagasaka, Oda, Iwatsubo, Kawamura, & Usui, 2012).

**Table 1.** Summary of drug-specific pharmacokinetic properties, side-effects and overdosing risks in women

	Pharmacokinetics			Risk for side-effects					Risk of overmedicating women as compared to men
	Metabolism^1^	CYP-activity in females as compared to males^2,3,4,5,6^	P-gp binding^7,8,9^	QTc prolongation	Prolactin elevation	EPS and akathisia^10^	Metabolic dysfunction		
							Weight gain^10^	Lipid/glucose abnormalities^12^	
Amisulpride	>90% renal excretion		*++/+++*	+++^10^	+++^10,12^	+^10^	+	++^12^	++
Aripiprazole	**CYP2D6, CYP3A4**	**(+), (+ +)**	++	**−**^10^	−^10,12^	−/+^10^	+^10^	−^12^	*+*
Chlorpromazine	CYP1A2*^a^*, CYP2D6	(− −), (+)	+	++^11ψ^	+^10ψ^	++^10ψ^	+++^10^	+++^12ψ^	++
Clozapine	**CYP1A2***^a^*, **CYP2C19***^a^,* CYP3A4	**(− −), (−),** (++)	+	++^11ψ^	−^10,12^	−^10^	+++^10^	+++^13^	+++
Flupentixol	**CYP2D6***^a^*	(+)	?	+^11ψ^	−^10^	+++^10^	++^10^	?	+
Haloperidol	**CYP2D6***^a^,* **CYP3A4**	**(+), (+ +)**	+	+^10ψ^	++^10.12^	+++^10^	+^10^	−^13^	+
Lurasidone	**CYP3A4**	(+ +)	?	**−**^10^	+/++^10,12^	++^10^	+^10^	−^13^	+/−
Olanzapine	**CYP1A2***^a^*	(− −)	++/+++	++^10^	+^10,12^	−^10^	+++^10^	+++^13^	+++
Paliperidone	CYP3A4, UGT1A1, 60% renal excretion	(++), (+)	*++/+++*	+^10^	+++^10.12^	+^10^	+^10^	++^13^	++
Quetiapine	**CYP3A4,** CYP2D6*^a^*	**(++)**, (+)	−/+	++^10^	−^10.12^	−^10^	+++^10^	++^13^	−
Risperidone	**CYP2D6***^a^*, CYP3A4	**(+)**, (++)	+++*	++^10^	+++^10.12^	++^10^	++^10^	+^13^	++
Sulpiride	Renal excretion only		++/+++	++	+++^12ψ^	++^10ψ^	++^10^	?	++
Zuclopenthixol	**CYP2D6**	**(+)**	?	?	?	+++^10^	+^10^	?	+

CYP, cytochrome P450; EPS, extrapyramidal symptoms; P-gp, P-glycoprotein; UGT, UDP glucuronosyl transferase.

The risk of overmedicating women as compared to men is estimated based on drug-specific metabolism, P-gp binding and CYP-activity in women compared to men. In addition, the risk of QTc prolongation, prolactin elevation, EPS and akathisia, weight gain and lipid/glucose abnormalities are also defined.

1, Hiemke et al. (2018); 2, Scandlyn et al. (2008); 3, Choi et al. (2013); 4, Piccinato et al. (2017); 5, Hagg et al. (2001); 6, Tamminga et al. (1999); 7, Doran et al. (2005); 8, Linnet & Ejsing (2008); 9, Nagasaka et al. (2012); 10, Huhn et al. (2019); 11, Wenzel-Seifert et al. (2011); 12, Peuskens et al. (2014); 13, De Hert et al. (2012).

(++), activity strongly higher in females; (+), activity higher in females; only of relevance during pregnancy; (−), activity lower in females; (− −), activity strongly lower in females; *a*, inhibited by oral oestrogenic contraceptives; +++, high incidence/high severity/strong; ++, moderate incidence/moderate severity/moderate; +, light incidence/mild severity/mild; −, low/very low/small; ?, unknown; *, main metabolite of risperidone 9-OH-risperidone. ψ, level of evidence is limited. When enzymes are indicated in bold, drug plasma concentrations will significantly increase or decrease when combined with strong to moderate inducers or inhibitors (see Hiemke et al., 2018).

Women naturally have more subcutaneous fat compared to men and this slows the absorption and perfusion of LAI antipsychotics (Soldin, Chung, & Mattison, 2011; Yonkers, Kando, Cole, & Blumenthal, 1992). The accumulation of LAI antipsychotics in adipose tissue increases their half-life time, resulting in extended release to the blood (Soldin et al., 2011; Yonkers et al., 1992). Short dosing intervals could lead to higher serum concentrations over time (Seeman, 2020). Female patients may, thus, benefit from longer dosage intervals for LAI compared to males.

### Metabolism

Drug metabolism is generally lower in the female sex, as women have a smaller liver and differential functioning of the hepatic cytochrome P450 (CYP) system compared to men (Soldin et al., 2011). Most antipsychotics are metabolised by CYP1A2, CYP2C19, CYP2D6 and/or CYP3A4 (Table 1) (Hiemke et al., 2018; Ravyn, Ravyn, Lowney, & Nasrallah, 2013). Of note, fluvoxamine has an inhibitory effect on all CYP enzymes, especially on CYP1A2 and CYP2C19, whereas paroxetine and fluoxetine are both potent inhibitors of CYP2D6 metabolism. Co-medication with these SSRIs may, thus, require downward dosage adjustments (Spina & De Leon, 2014).

#### CYP1A2

CYP1A2 activity is lower in females compared to that in males (Scandlyn, Stuart, & Rosengren, 2008). Clozapine and olanzapine are both mainly metabolised by this enzyme and with similar dosing, these antipsychotics reach 25–60% higher dose-adjusted concentrations in women (Bigos et al., 2008; Castberg et al., 2017; Jönsson et al., 2019; Tang et al., 2007). Since both oestrogen and progesterone are substrates of CYP1A2, they have an inhibitory effect on this enzyme (Lu et al., 2020). Drug plasma concentrations are, therefore, specifically higher in the female sex when hormone levels are high (e.g. before menopause) whereas they decrease in relative terms when hormone levels fall (e.g. after menopause) (Soldin et al., 2011).

#### CYP3A4

In contrast to CYP1A2, CYP3A4 activity is about 20–30% higher in premenopausal women as compared to the other sex (Greenblatt & von Moltke, 2008; Scandlyn et al., 2008; Wolbold, 2003; Yang & Li, 2012) and its activity is stimulated by oestrogens and progesterone (Choi, Koh, & Jeong, 2013; Piccinato et al., 2017). CYP3A4 metabolism is especially relevant for female patients taking quetiapine and lurasidone since these drugs are only metabolised by this enzyme (Table 1). For CYP3A4 substrates, the previously discussed plasma concentration elevating processes are neutralised by this higher CYP3A4 activity, some of which was demonstrated in a large sample of adult patients (Jönsson et al., 2019). Jönsson et al. (2019) found smaller sex differences in dose-adjusted concentrations for partial CYP3A4 substrates aripiprazole (*n* = 1610) and haloperidol (*n* = 390) than for CYP1A2 substrate olanzapine (*n* = 10 286), and even lower dose-adjusted concentrations for quetiapine (*n* = 5853). Moreover, Castberg et al. (2017) showed that the blood levels of quetiapine (*n* = 4316) are similar in males and females before menopause; whereas after menopause, quetiapine concentrations are higher in females compared to that in males.

#### CYP2D6

Aripiprazole, haloperidol and risperidone are some of the antipsychotics that are primarily metabolised by CYP2D6 (Table 1). According to a population-based study (*n* = 1376, 23% females, 18–82 years), CYP2D6 activity is 20% higher in women as compared to men (Tamminga et al., 1999). The clinical relevance of this sex difference in CYP2D6 activity is probably small (Aichhorn et al., 2005), as individual differences largely depend on highly variable genetic variations in the CYP2D6 gene (Labbé et al., 2000). Sex differences may, therefore, only become apparent when female sex hormones reach significant heights (Gaedigk, Dinh, Jeong, Prasad, & Leeder, 2018; Konstandi, Andriopoulou, Cheng, & Gonzalez, 2020), for example in pregnancy.

#### CYP219C

CYP219C is responsible for the metabolism of clozapine and other less commonly used antipsychotics (e.g. cyamemazine) (Hiemke et al., 2018). The population-based study by Tamminga et al. (1999) (*n* = 2638, 30% females, 18–82 years) reported a 40% lower enzyme activity in females compared to that in males, which was most pronounced in the age range from 18 to 40 years. Yet, these findings were not replicated in a sample of 330 healthy individuals (*n* = 611, 18–49 years) (Hagg, Spigset, & Dahlqvist, 2001).

### Excretion

The three major processes that determine renal clearance [i.e. glomerular filtration rate (GFR), tubular absorption and tubular excretion] are all lower in women compared to that in men (Soldin et al., 2011). Since P-gp facilitates renal drug efflux, lower P-gp levels lead to slower renal and hepatic clearance in women as compared to men (Elmeliegy et al., 2020; Nicolas et al., 2009). After adjusting for body size, GFR is 10–25% lower in females (Whitley & Lindsey, 2009). These differences in renal clearance are particularly relevant for amisulpride, sulpiride and paliperidone, which are cleared mainly by the kidney, leading to much higher plasma levels of these drugs in female patients of all ages (Hoekstra et al., 2021; Li, Li, Shang, Wen, & Ning, 2020).

## Differences in pharmacodynamics between men and women

Oestrogens can modulate the effect of dopamine (Gogos et al., 2015; Shams, Sanio, Quinlan, & Brake, 2016; Yoest, Cummings, & Becker, 2019). According to preclinical evidence, oestrogens influence the levels of dopamine transporters (DATs) and receptors in cortical and striatal regions and by increasing D2 receptor sensitivity in the ventral tegmentum (Vandegrift, You, Satta, Brodie, & Lasek, 2017). A single photon emission computerised tomography DAT study showed an effect of sex (effect size: 0.25), whereby female patients with schizophrenia had a higher ratio of specific striatal binding than male patients (17–53 years) (Chen et al., 2013). These sex differences in the organisation of the striatal dopamine system result in higher D2 receptor occupancy in premenopausal women compared to that in men, even when plasma concentrations are similar, whereas postmenopausal women need higher dosages to reach the same efficacy (Fig. 1).

## Differences in treatment response between men and women

Meta-analyses on the efficacy of antipsychotic drugs often do not take sex into account (Huhn et al., 2019; Leucht et al., 2020). Yet, based on the limited number of studies that did account for sex, a better response in women has been reported for most antipsychotic drugs (Ceskova et al., 2015; Lange et al., 2017; Usall, Suarez, & Haro, 2007), except for amisulpride (Ceskova et al., 2015; Kahn et al., 2018; Müller et al., 2006), risperidone (Ceskova et al., 2015; Labelle, Light, & Dunbar, 2001; Pu et al., 2020; Segarra et al., 2012; Usall et al., 2007) and perhaps clozapine (Alberich et al., 2019). As premenopausal women overall show better treatment response and fewer hospitalisations compared to postmenopausal women (Ayesa-Arriola et al., 2020; Goldstein et al., 2002; Seeman, 2019; Shlomi Polachek et al., 2017), inconsistent findings may be a result of heterogeneity in age and in duration and severity of illness between study samples. However, all of the abovementioned studies on antipsychotic efficacy fail to account for differences in dose-adjusted plasma concentrations, which may have confounded their results. Specifically, the pronounced sex differences in drug pharmacokinetics and D2 binding rates result in much higher plasma levels in women and especially premenopausal women compared to that in men, even when dose adjustments for body weight are performed (Jönsson et al., 2019).

The augmentative effect of oestrogens on treatment efficacy is also evident in other patient populations. For example, female patients with anorexia nervosa receiving antipsychotics may benefit from oestrogen augmentation therapy, as these women also have low oestrogen levels (Keating, 2010). In addition, when women are more sensitive to antipsychotics, they would also be more sensitive to other forms of dopaminergic medications. Levodopa is a precursor of dopamine and is the most potent medication for Parkinson's disease (PD). Indeed, levodopa appears to be more effective at reducing symptoms in female compared to that in male patients with PD (Lyons, Hubble, Tröster, Pahwa, & Koller, 1998), whereas female sex is also indicated as a risk factor for levodopa-induced side-effects, such as dyskinaesia and hallucinations (Hassin-Baer et al., 2011; Zhu, van Hilten, Putter, & Marinus, 2013).

## Side-effects and tolerability

Although higher plasma levels increase bioavailability and efficacy, they also increase the risk for adverse events (Fig. 1) (Castberg et al., 2017; Jönsson et al., 2019; Lange et al., 2017). Indeed, a large study of 1087 patients between 18 and 65 years with psychotic disorders (48% female) shows that female gender was one of the two main risk factors for severe side-effects, together with polypharmacy, which is also more common in women (Iversen et al., 2018).

### QTc-prolongation

Prolongation of the QTc interval is a side effect of several antipsychotics and may result in life threatening cardiac ventricular arrhythmia such as torsades de pointes (TdP) (Beach, Celano, Noseworthy, Januzzi, & Huffman, 2013). QTc intervals are typically longer in women compared to that in men and female sex is an independent risk factor for developing drug-related TdP (De Yang et al., 2011; Makkar, 1993). Although testosterone appears to shorten the QTc interval in men, there seems to be a more complex interaction between progesterone and oestrogen in women (Vink, Clur, Wilde, & Blom, 2018). De Yang et al. (2011) evaluated electrocardiograms of 1006 schizophrenia patients (32% female, 25–75 years) on typical and atypical antipsychotic medication and reported that QTc prolongation was more than twice as common in females (7.3%) compared to that in males (3.2%). Although most antipsychotics can cause QTc prolongation, amisulpride, risperidone, olanzapine and quetiapine have the highest risk (Table 1) (Huhn et al., 2019). Elderly women are more vulnerable for developing TdP as a consequence of QTc prolongation, since sex hormone levels decline whereas additional risk factors for TdP, such as heart disease and electrolyte changes, become more common with increasing age (Danielsson et al., 2016; Wenzel-Seifert, Wittmann, & Haen, 2011). Clinicians should, thus, be wary of the increased risk of QTc prolongation in (older) women, especially those with a (family) history of heart disease.

### Extrapyramidal side-effects

Antipsychotics such as amisulpride, risperidone, paliperidone and aripiprazole are associated with a high risk of extrapyramidal symptoms (EPSs) (Huhn et al., 2019). Although acute dystonia appears to be less common in premenopausal female patients (Mas et al., 2016), women seem to have a greater risk of developing parkinsonism and akathisia compared to males (Divac, Prostran, Jakovcevski, & Cerovac, 2014), which is likely due to the higher dopamine receptor binding at lower dosages in women (Di Paolo, 1994; Seeman & Lang, 1990; Thanvi & Treadwell, 2009). The higher risk for women of developing parkinsonism has also been reported in other patient populations that are prescribed antipsychotic medications, for example in patients with dementia (Marras et al., 2012). Multiple reviews indicate that an increased prevalence of EPS in women is associated with a postmenopausal oestrogen decline (da Silva & Ravindran, 2015; Leung & Chue, 2000; Seeman & Lang, 1990; Thompson, Kulkarni, & Sergejew, 2000). Long-term exposure to dopamine receptor-blocking agents can cause tardive dyskinaesia (TD), which is also more common in women compared to that in men of all ages (Divac et al., 2014; Yassa & Jeste, 1992). The incidence of antipsychotic-induced TD is relatively low in premenopausal women (3–5%), possibly due to the protective antioxidant effects of oestrogens (Cho & Lee, 2013; Wu, Kosten, & Zhang, 2013), but can reach an incidence rate of 30% in postmenopausal women after 1 year of cumulative exposure to antipsychotics (Waln & Jankovic, 2013).

### Endocrine side-effects

By diminishing the inhibitory effect of dopamine on prolactin secretion in the pituitary gland, antipsychotics often cause hyperprolactinaemia (González-Rodríguez, Labad, & Seeman, 2020), which can result in galactorrhoea, cessation of normal cyclic ovarian function and hirsutism (Malik et al., 2011; Peuskens, Pani, Detraux, & De Hert, 2014). Premenopausal women have physiologically higher levels of prolactin compared to men and are therefore closer to the threshold for hyperprolactinaemia (Kaar, Natesan, McCutcheon, & Howes, 2020; Riecher-Rössler, 2017). Consequently, they are more than twice as likely to develop antipsychotic-induced hyperprolactinaemia compared to postmenopausal women and men (González-Rodríguez et al., 2020; Kinon, Gilmore, Liu, & Halbreich, 2003). Moreover, prolactin secretion suppresses the production of sex hormones and induces oestrogen deficiency, which is already more frequent in female SSD patients compared to healthy females before menopausal age (Brand et al., 2021; Gogos et al., 2015; Lindamer et al., 2000; Riecher-Rössler, 2005). Oestrogen deficiency can lead to polycystic ovarian syndrome, infertility, osteoporosis, sexual dysfunction and an increased risk of breast cancer (De Hert, Detraux, & Peuskens, 2014; Haring et al., 2014; Pottegård, Lash, Cronin-Fenton, Ahern, & Damkier, 2018; Yum, Kim, & Hwang, 2014). For example, up to 48% of women receiving antipsychotic treatment report irregularities in their menstrual cycle (O'Keane, 2008) and reduced bone mineral density is present in 32% of women treated with prolactin-raising antipsychotics for >10 years (Meaney et al., 2004). Prolactin-sparing antipsychotics (e.g. aripiprazole) should, therefore, be preferred over prolactin-raising antipsychotics (e.g. risperidone) for female patients of all ages (Table 1).

### Metabolic side-effects and risk for cardiovascular disease

Women treated with antipsychotics are 1.7 times more likely to develop metabolic syndrome compared to men and are therefore at a higher risk for cardiovascular disease (CVD) (Bener, Al-Hamaq, & Dafeeah, 2014; Carliner et al., 2014; Huang et al., 2009; Ingimarsson, MacCabe, Haraldsson, Jónsdóttir, & Sigurdsson, 2017; McEvoy et al., 2005). When taking antipsychotics, women are specifically more vulnerable to weight gain and abdominal adiposity (up to 73.4% in women *v.* 36.6% in men) (Alberich et al., 2019; Kraal, Ward, & Ellingrod, 2017; McEvoy et al., 2005; Panariello, De Luca, & de Bartolomeis, 2011; Verma, Liew, Subramaniam, & Poon, 2009), which is worrisome as abdominal obesity is a strong predictor of diabetes (de Vegt et al., 2001). Increased appetite and food intake can be a result of the antagonistic effect of antipsychotics on several neurotransmitter systems (i.e. serotonergic, histaminergic and dopaminergic) (Bak, Fransen, Janssen, Van Os, & Drukker, 2014). Since most antipsychotics are given at doses higher than required in women (Jönsson et al., 2019), they are more vulnerable to gain weight from overeating, as high drug plasma levels lead to higher receptor occupancy and thus induce more appetite. Strong associations are particularly found between weight gain and histamine H1 antagonism (Vehof et al., 2011). Since oestrogens modulate histamine neurotransmission (Provensi, Blandina, & Passani, 2016), the antagonistic action of antipsychotics on this receptor may also be enhanced by oestrogens, which could explain why the effects on appetite and food intake are stronger in premenopausal women compared to that in postmenopausal women and men. Special attention should be paid to clozapine and olanzapine, which both have the highest affinity for the histamine H1 receptor and the highest risk of being prescribed at doses higher than the required. Unsurprisingly, these antipsychotics are also the ones most strongly associated with weight gain (Kaar et al., 2020; Smith, Leucht, & Davis, 2019). Adequate dosing of all antipsychotics is, therefore, crucial (Huhn et al., 2019; Kraal et al., 2017), and medications that induce minimal weight gain like aripiprazole, may be preferred (Table 1).

Oestrogen deficiency in women can induce several metabolic symptoms, including insulin resistance and hypertriglyceridaemia (Fitzgerald, Janorkar, Barnes, & Maranon, 2018; Valencak, Osterrieder, & Schulz, 2017). The risk of metabolic dysfunction and CVD is, thus, increased by all factors that cause oestrogen deficiency (e.g. hyperprolactinaemia and menopause) (Regitz-Zagrosek, Lehmkuhl, & Mahmoodzadeh, 2007). Moreover, low oestrogen levels cause abdominal fat accumulation, since oestrogens augment fat accumulation around the hips and thighs rather than upper abdominal areas (Fitzgerald et al., 2018). This again highlights the importance of avoiding prolactin-raising antipsychotics in women to prevent hyperprolactinaemia.

## Special considerations regarding pharmacotherapy in premenopausal women

Since pharmacokinetic processes are generally lower in women whereas D2 occupancy rates are higher during high oestrogen phases, dosing of most antipsychotics should start lower in premenopausal women than in men, except for quetiapine and lurasidone. At times when oestrogen levels are high (i.e. during the ovulatory phase), premenopausal women are especially vulnerable to overdosing of drugs metabolised by the CYP1A2 enzyme (olanzapine and clozapine) (Table 1). Although further research is required, it is possible that premenopausal women may, on average, have enough antipsychotic protection from a dose of clozapine and olanzapine that is half of an average male dose (Eugene & Masiak, 2017). The interaction between antipsychotics and the menstrual cycle is subject to individual differences and requires further investigation for each type of antipsychotic. In general, women with regular cycles who suffer from psychotic exacerbations during the menstrual phase may benefit from slight dose increments shortly before and around menstruation, although this is rarely done in practice (González-Rodríguez & Seeman, 2019; Lange et al., 2017; Seeman, 2020; Yum, Yum, & Kim, 2019). Side-effects of antipsychotic drugs that could be particularly relevant to younger female patients include weight gain, as obesity largely decreases self-esteem in women (Connors & Casey, 2006; Lieberman, Tybur, & Latner, 2012) and hyperprolactinaemia, which reduces oestrogen levels. These low oestrogen levels not only increase the risk for somatic complications but also potentially have also a negative impact on psychotic symptoms (Brand et al., 2021). Aripiprazole and lurasidone may, therefore, be drugs of first choice in this phase and options of second choice are olanzapine, quetiapine and clozapine (Table 2).

**Table 2.** Summary of clinically relevant treatment considerations across different hormonal phases

		Premenopausal women	Pregnant women	Postpartum/breastfeeding women	Postmenopausal women
First choice		Aripiprazole, lurasidone	Quetiapine, haloperidol, olanzapine	Olanzapine	Aripiprazole, lurasidone
Second choice		Olanzapine, quetiapine, clozapine	Aripiprazole, lurasidone	Amisulpride, haloperidol (when breastfeeding)	Olanzapine, quetiapine, clozapine
Avoid		Prolactin-raising antipsychotics	Polypharmacy; risperidone, although switching is not recommended	Clozapine (when breastfeeding)	Sertindole, amisulpride, prolactin-raising antipsychotics
Starting dose		Should be generally lower than in men, but similar for quetiapine and lurasidone	Minimum effective dose	Similar dose as before pregnancy	Dose increase after menopause
Monitor		Metabolic complications	Gestational diabetes, (oral) glucose tolerance testing, cholesterol and triglycerides	Neurodevelopment and motor abnormalities in baby	Metabolic complications
		Sex hormone/prolactin levels, especially when using prolactin-raising antipsychotics	Drug plasma levels, particularly in third trimester	Maternal clinical symptoms	Sex hormone/prolactin levels, especially when using prolactin-raising antipsychotics
Special considerations	General	Be wary of different menstrual phases and associated fluctuations in plasma concentrations	Switching from usual prescription is not recommended	Continue antipsychotic treatment postpartum	Consider augmentation with SERM raloxifene
		Oestrogenic contraceptives strongly increase clozapine levels: progesterone-only contraceptives or frequent monitoring of plasma levels is recommended	The potential benefits of antipsychotics may outweigh their potential risks of discontinuation	Limited amount of evidence on the effects on the baby. Possible antipsychotic use during breastfeeding should be considered in discussion with patient, partner or carer, and paediatrician as required	
	Dosing	Consider slight increases (e.g. 10%) in dosing shortly before and during menstruation in women with regular cycles. These dose adjustments are dependent of drug-specific P-gp affinity and metabolism	Plasma levels may change significantly in third trimester and plasma levels of renally excreted drugs decrease	Dose increments may be required to prevent postpartum psychotic exacerbations	Dose adjustments are dependent on drug-specific P-gp affinity and metabolism Doses should be higher for most antipsychotics compared to the premenopausal period, except maybe for quetiapine and lurasidone, which may even be lowered
	Side-effects	Be wary of risk of weight gain and hyperprolactinaemia	Increased risk of gestational diabetes mellitus, except for aripiprazole	Limited amount of evidence on the effects of breastfeeding on the baby	Be wary of increased risk of QTc prolongation, motor symptoms (parkinsonism, akathisia and TD), CVD and osteoporosis

### Interaction between antipsychotics and contraceptives

Women with SSD are usually prescribed contraceptives to decrease the probability of psychotic exacerbations during (pre)menstrual phases of the cycle. Considering the protective effect of oestrogens on psychosis, oestrogenic contraceptives may be preferred over progesterone-only contraception (Brand et al., 2021). Yet, oestrogenic contraceptives also increase the risk of CVD (McCloskey et al., 2021; Seeman & Ross, 2011), making progesterone-only contraceptives (e.g. long-acting reversible contraceptives or intrauterine devices) preferred methods, especially in women over the age of 35 or in those who smoke or who suffer from hypertension (Curtis et al., 2016). Oestrogenic contraceptives inhibit CYP1A2 and CYP2C19 activity, increasing plasma concentrations of their substrates (Table 1) (Granfors, Backman, Lattila, & Neuvonen, 2005; Hagg et al., 2001; Hiemke et al., 2018; Ramsjö et al., 2010; Tamminga et al., 1999). For clozapine, plasma concentrations may increase two- to three-fold in the active hormone phase compared with the no-hormone phase, which may result in significant side-effects such as hypotension, sedation and sialorrhoea (Table 2) (Bookholt & Bogers, 2014; McCloskey et al., 2021). Progesterone-only contraceptives may be preferable for women treated with clozapine (McCloskey et al., 2021). Alternatively, frequent monitoring of plasma levels is recommended when using oestrogenic contraceptives, since oestrogenic birth control may have beneficial effects on the symptoms of SSD (Brand et al., 2021).

It should be noted that there is paucity in studies on this subject; therefore, the effects of hormonal contraceptives on antipsychotic plasma concentration are not clear for other antipsychotic drugs besides clozapine. Based on research so far, it seems there are no clinically relevant changes in the other antipsychotics when combined with contraceptives, although some studies suggest small changes in olanzapine plasma levels (Berry-Bibee et al., 2016).

## Special considerations regarding pharmacotherapy in pregnant women

Women who take antipsychotics are generally at a higher risk of adverse maternal and infant outcomes including congenital malformations, preterm birth, foetal growth abnormalities and poor neonatal adaptation. Compared to healthy, unexposed pregnant women, these women are also more likely to have poor living conditions, poor life-style habits (e.g. smoking, drinking and bad eating habits) and metabolic dysfunctions (McAllister-Williams et al., 2017; Terrana, Koren, Pivovarov, Etwel, & Nulman, 2015). According to studies that control for these factors, antipsychotics themselves are not significantly associated with an increased risk of stillbirth, small/large-for-gestational-age births, preterm delivery or spontaneous miscarriage (Beex-Oosterhuis et al., 2020; Boden, Fergusson, & Horwood, 2011; Cuomo, Goracci, & Fagiolini, 2018; Damkier & Videbech, 2018; Ennis & Damkier, 2015; Huybrechts et al., 2016; Petersen et al., 2016; Reinstein, Cosgrove, Malekshahi, & Deligiannidis, 2020; Tosato et al., 2017; Vigod, Gomes, Wilton, Taylor, & Ray, 2015). Of note, risperidone has been associated with a higher risk for congenital malformations (relative risk of 1.26) (Huybrechts et al., 2016). Quetiapine, haloperidol and olanzapine are the most frequently investigated antipsychotics in pregnancy, which may be the reason why they are most commonly prescribed during this period (Gentile, 2010; Hasan et al., 2015; McAllister-Williams et al., 2017). However, current knowledge on the foetal risks of individual antipsychotics is limited and based on non-randomised studies or case series reports, making it challenging to draw firm conclusions (Damkier & Videbech, 2018; McAllister-Williams et al., 2017). In general, the potential benefits of antipsychotics may outweigh their potential risks of discontinuation. Switching a woman before or during pregnancy from their usual prescription may negatively affect clinical course and well-being. Careful consideration should, thus, guide clinical decisions in women with a diagnosis of SSD at this vulnerable time. In addition, polypharmacy, especially with SSRIs or mood stabilisers should be avoided, as it has been associated with more complications during pregnancy (Table 2) (Sadowski, Todorow, Yazdani Brojeni, Koren, & Nulman, 2013). Although lithium is considered to be relatively safe, valproate and carbamazepine are associated with major congenital malformations (Grover & Avasthi, 2015).

Pregnant women taking antipsychotics have been reported to have up to a 20.9% higher risk of developing gestational diabetes mellitus, especially when using olanzapine, risperidone, clozapine or high doses of quetiapine (>300 mg/day) (Galbally, Frayne, Watson, Morgan, & Snellen, 2020). Two meta-analyses on this subject confirm this higher risk, although evidence remains insufficient due to significant heterogeneity across studies (Kucukgoncu et al., 2019; Wang et al., 2021). Pregnant female patients should, thus, be monitored to prevent metabolic complications, with glucose tolerance testing and assessment of cholesterol and triglycerides (Table 2) (Breadon & Kulkarni, 2019).

### Dosing during pregnancy

Physiological changes during pregnancy include increases in plasma volume, drug elimination rates and sex hormone levels (Payne, 2019). The activity of albumin and P-gp decrease by 31% and 19%, respectively, in late pregnancy (Abduljalil, Furness, Johnson, Rostami-Hodjegan, & Soltani, 2012; Ke, Rostami-Hodjegan, Zhao, & Unadkat, 2014), whereas the blood–brain barrier permeability increases, together leading to higher drug bioavailability. Moreover, renal excretion increases (Segarra et al., 2020), which results in lower plasma concentrations of amisulpride, sulpiride and paliperidone (Ke et al., 2014). CYP2D6 and CYP3A4 activity increases up to 50–100% in the third trimester while CYP1A2 and CYP2C19 activity decreases to up to 40–50% (Choi et al., 2013; Hiemke et al., 2018; Payne, 2019) (Table 1). Although research on this topic is limited, these metabolic changes appear to have significant consequences on the plasma levels of CYP3A4/CYP2D6 substrates quetiapine (in 35 pregnancies) and aripiprazole (in 14 pregnancies), which were found to be reduced by more than 50% in the third trimester whereas plasma levels of olanzapine (in 29 pregnancies) and clozapine (in 4 pregnancies) were found to be unchanged (Westin et al., 2018). For clozapine, there is some evidence for increased serum levels in the third trimester (Nguyen, Mordecai, Watt, & Frayne, 2020). Summarising, drug monitoring and blood level assessment is required throughout pregnancy and becomes increasingly important in the third trimester (Table 2) (Dazzan, 2021).

## Special considerations regarding pharmacotherapy in postpartum women

After birth and especially in the first few weeks after delivery, women with bipolar disorder, schizoaffective disorder, or a history of postpartum psychosis are at an increased risk of postpartum psychosis due to hormonal alterations, neuro-immune changes and psychosocial factors (Hazelgrove et al., 2021; Meltzer-Brody et al., 2018). In these women, it is important to closely monitor clinical symptoms in this period and to consider continuation of antipsychotic treatment (Jones, Chandra, Dazzan, & Howard, 2014). Of note, pharmacological agents that suppress lactation are usually dopamine D2 agonists and are suggested to increase the risk of postpartum psychosis. This type of medication should, therefore, not be prescribed in postpartum women with SSD (Snellen, Power, Blankley, & Galbally, 2016).

### Breastfeeding

Although breastfeeding has clear benefits for bonding and immunity (Chandra, Babu, & Desai, 2015), women with SSD are currently encouraged to breastfeed, unless they are taking clozapine (NICE, 2014). Although all antipsychotics are excreted into the breastmilk, the amount of active ingredients that is transferred to the infant is relatively low (Pacchiarotti et al., 2016). Based on a systematic review of 49 studies, the highest penetration ratio has been found for amisulpride, followed by clozapine and haloperidol (Schoretsanitis, Westin, Deligiannidis, Spigset, & Paulzen, 2020). Anecdotal evidence on clozapine has reported considerable concentrations in breast milk, risk of agranulocytosis in the infant and potentially delayed speech development low (Pacchiarotti et al., 2016). Based on the current literature, positive safety data are most consistent for olanzapine (Zincir, 2019). Apart from clozapine, other common antipsychotics are rarely associated with adverse events (Uguz, 2016), but monitoring of neurodevelopment and motor abnormalities is warranted due to the limited amount of evidence (McAllister-Williams et al., 2017; NICE, 2014). Taken together, the choice of feeding method and about which antipsychotic is best taken is difficult and should be part of a shared decision process involving the partner and paediatrician (Table 2).

## Special considerations regarding pharmacotherapy in postmenopausal women

Most women require a higher dose of antipsychotic medication after menopause, when oestrogen levels decline and the sensitivity to dopamine reduces. In this phase of life, symptoms often increase and menopausal complaints such as sleep disturbances and mood swings can increase the risk of psychotic relapse (Brand et al., 2021; Riecher-Rössler, 2017). These dose increments are dependent on drug metabolism. Moreover, dose increments may be smaller or even redundant for lurasidone and quetiapine, since plasma levels of these antipsychotics increase significantly when oestrogen levels decrease.

Amisulpride and sertindole are not recommended as they induce QTc prolongation and typical antipsychotics or risperidone are not the best choice either, as they induce motor symptoms for which older women are more vulnerable (Table 1) (Lange et al., 2017; Leung & Chue, 2000). Clinicians should also be wary of drugs more likely to induce metabolic dysfunction and hyperprolactinaemia (Seeman, 2012). This leaves aripiprazole and lurasidone as potential drugs of first choice for postmenopausal women. Quetiapine has a higher risk for certain side-effects and can therefore be drug of second choice. Olanzapine or clozapine are other possible alternatives, as they are associated with higher risks for side-effects as well (Table 2), but may also be more effective than aripiprazole, quetiapine and lurasidone (Huhn et al., 2019).

Oestrogen augmentation should be considered at an early stage (i.e. at the beginning of menopause, or even earlier) as this can improve antipsychotic response, reduce psychotic symptoms and relief menopausal complaints (e.g. mood and sleep disturbances and osteoporosis) (de Boer, Prikken, Lei, Begemann, & Sommer, 2018; Heringa, Begemann, Goverde, & Sommer, 2015). As oestrogen replacement also increases bone mineral density, the prevention of antipsychotic-induced osteoporosis is a secondary benefit. Selective oestrogen receptor modulators (SERMs) such as raloxifene are more suitable for long-term use compared to oestrogens, since SERMs have oestrogenic effects on the brain and bone tissue but anti-oestrogenic effects on other tissues (such as breast and uterus) (Arevalo, Azcoitia, & Garcia-Segura, 2015). As hormone replacement therapy with either oestrogens or SERMs is associated with increased risk of thromboembolic events (Ellis, Hendrick, Williams, & Komm, 2015), potential benefits and disadvantages should be balanced individually. For example, a (family) history of thrombo-embolic events may be a reason not to opt for oestrogen replacement therapy.

## Conclusion

Women tend to have slower drug absorption, distribution, metabolism and elimination rates, resulting in higher plasma levels and bioavailability of most antipsychotic medications. Since oestrogens induce dopamine sensitivity in the brain, the efficacy of antipsychotics is enhanced in premenopausal women, compared to men. Considering that current treatment guidelines are mostly based on data from men, women are likely to be overmedicated and it is therefore not surprising that adverse events are much more common in women. Additionally, women are more vulnerable to many side-effects, such as weight gain, EPS and hyperprolactinaemia, independently of dose. Defining the minimum effective dose in women is of utmost importance, as the risk of overmedicating increases when female sex hormone levels are high, specifically during ovulation and in the later stages of pregnancy. Conversely, effective doses of most antipsychotics (quetiapine and lurasidone are exceptions) may need to be increased during low-hormonal phases in life, specifically post-natal, during menstruation and during and after menopause. Optimal antipsychotic treatment for women is highly dependent on the different life phases. Premenopausal women should be prescribed lower dosages for most antipsychotics (except for quetiapine and lurasidone). During pregnancy, the most reasonable and less harmful choice appears to be maintaining future mothers with SSD at the minimum effective dosage of the drug they were already using before pregnancy. If a medication-free pregnancy is feasible, this is of course the best option. Clozapine should probably be avoided during breastfeeding and while further safety data on other antipsychotics are limited, the inherent benefits of breastfeeding for mother and baby should be balanced carefully against the potential risks for the baby. Postmenopausal women represent a group of patients that is especially vulnerable to side-effects associated with ageing and declining hormone levels such as osteoporosis and CVD, and for these women, oestrogen replacement therapy should be considered to ameliorate both somatic and mental health problems, although the increased risk for thrombosis also needs to be taken into account.
