# Antipsychotic medication for women with schizophrenia spectrum disorders – CORRIGENDUM

**DOI:** 10.1017/S0033291723001344

**Published:** 2023-07

**Authors:** Bodyl A. Brand, Yudith R. A. Haveman, Franciska de Beer, Janna N. de Boer, Paola Dazzan, Iris E. C. Sommer

**Affiliations:** 1 Department of Biomedical Sciences of Cells & Systems, Section Cognitive Neurosciences, University Medical Center Groningen, University of Groningen, Groningen, the Netherlands; 2 Department of Psychiatry, UMC Utrecht Brain Center, University Medical Center Utrecht, Utrecht University, Utrecht, the Netherlands; 3 Department of Psychological Medicine, Institute of Psychiatry, Psychology and Neuroscience, King's College London, London, UK; 4 National Institute for Health Research (NIHR) Mental Health Biomedical Research Centre at South London and Maudsley NHS Foundation Trust and King's College London, London, UK

When this article was originally published in Psychological Medicine it included the sentence ‘'Women's stomachs are on average less acidic compared to men's and this increases the absorption of antipsychotic drugs’. Based on the available literature on this topic, it is difficult to determine whether, and to what extent, sex differences in stomach acidity influence the absorption of antipsychotics. Due to this, this sentence has been removed and Figure 1 has been updated to reflect this change.

The authors apologise for this error.
